# Evaluation of the effect of *Loigolactobacillus coryniformis* K8 CECT 5711 consumption in health care workers exposed to COVID-19

**DOI:** 10.3389/fnut.2022.962566

**Published:** 2022-08-03

**Authors:** Raquel Rodriguez-Blanque, Juan Carlos Sánchez-García, Ángel Cobos-Vargas, Ana Aguilar Quesada, Jose A. Maldonado-Lobón, Mónica Olivares, Ruth Blanco-Rojo

**Affiliations:** ^1^Hospital Universitario San Cecilio, Granada, Spain; ^2^Department of Nursing, Faculty of Health Sciences, University of Granada, Granada, Spain; ^3^Hospital Virgen de las Nieves, Granada, Spain; ^4^Department of Research and Development, Biosearch Life, a Kerry Company, Granada, Spain

**Keywords:** probiotic, immune response, COVID-19, health care workers, randomized clinical trial

## Abstract

Following the spread of the SARS-CoV-2 coronavirus, an unprecedented burden has been placed on health care systems, with health care workers (HCWs) being most at risk of COVID-19 infection. The effect of the probiotic *Loigolactobacillus coryniformis* K8 CECT 5711 on frontline HCWs exposed to the virus was studied in a randomized, double-blind, placebo controlled trial. Parameters related to the incidence and severity of COVID-19 as well as the immune response and the side effects of the COVID-19 vaccine were evaluated. For 2 months, a group of 250 front-line HCWs over the age of 20 was randomly allocated to receive either *L. coryniformis* K8 or a placebo daily. SARS-CoV-2 infection incidence was verified via PCR or antigen test. In those volunteers who were vaccinated during the intervention, serum levels of specific IgG were analyzed at the end of the study. The incidence of COVID-19 infection was very low [IR (SD) = 0.016 (0.011)], and no significant difference was found between the groups [IRR (95% CI): 1.008 (0.140–7.268), *p* = 0.994]. For immune response analysis, the total sample was divided according to the days between the first dose and the antibody analysis (cutoff points were set at ≤ 56, 57–80 and ≥ 81 days). The specific IgG level decreased over time (*p* > 0.001). However, in the subgroup of subjects for whom more than 81 days had passed since they received the first dose, the specific IgG levels were significantly higher in the those that took the *L. coryniformis* K8 [7.12 (0.21)] than in the control group [6.48 (0.19)] (*P* = 0.040). Interestingly, the subjects who started probiotic consumption before the first dose reported significantly fewer side effects (of any kind) at the 1st dose of the vaccine (OR: 0.524, *p* = 0.043), specifically less arm pain (OR: 0.467, *p* = 0.017). In conclusion, the administration of *L. coryniformis* K8 CECT 5711 to HCWs helps to extend the immune protection generated by the COVID-19 vaccine over time.

## Introduction

The coronavirus disease 2019 (COVID-19), caused by the severe acute respiratory syndrome coronavirus 2 (SARS-CoV-2), has quickly spread throughout the world, leading to an enormous strain on health-care systems and inflicting millions of infections and deaths worldwide (1).

Health care workers (HCWs) are at high risk for COVID-19 infection, not only due to close contact with highly infectious patients, but also due to undiagnosed or subclinical infectious case exposure. Moreover, the stress and physical overexertion caused by the pandemic may affect the immune response of HCWs (2). Indeed, a meta-analysis of ninety-seven studies estimated that the prevalence of SARS-CoV-2 infection in HCWs was 11% up to July 2020 (3), which constituted a significant proportion of all COVID-19 patients (4). Although the severity and mortality among HCWs were relatively low compared to those of other population groups (3), these COVID-19 events have led in a shortage of health staff adding a burden in the pandemic fighting.

Because of their immunomodulatory, anti-inflammatory, antioxidant, and antiviral properties, some authors have suggested that probiotics may have a role in the prevention and/or moderation of COVID-19 severity (5–7). However, to date, only one study that has been performed in COVID-19 patients showed that a probiotic formula may help to reduce duration of digestive and non-digestive symptoms compare to placebo (8), so more studies were needed to determine the role of probiotics in this disease. In this sense, the *Loigolactobacillus coryniformis* K8 CECT 5711 strain has been demonstrated to present immunomodulatory activity in adults (9) and children (10, 11). Furthermore, when given orally to healthy individuals in the context of hepatitis A vaccination, this strain was demonstrated to boost specific antibody levels against the hepatitis A virus (12). Additionally, in another trial carried out in elderly participants, *L. coryniformis* K8 increased the immunological response to influenza vaccination and reduced the symptoms associated with respiratory infections (13). Very recently, a randomized clinical trial performed in COVID-vaccinated nursing home residents, evidenced the usefulness of *L. coryniformis* K8 in the context of the COVID-19 pandemic by increasing the specific immune response after infection with COVID-19 and by helping the vaccine-specific responses in the elderly (14).

Here, we report the findings of a randomized, placebo-controlled, double-blind trial to assess the effect of the consumption of the probiotic strain *L. coryniformis* K8 CECT 5711 on the incidence and severity of COVID-19 in frontline HCWs exposed to the virus. Additionally, the immune response and the side effects of the COVID-19 vaccine were evaluated in a subgroup of these HCWs.

## Materials and methods

### Study design and subjects

A randomized, double-blinded, placebo-controlled multicenter study was performed. The study was carried out in two of the reference hospitals treating COVID-19 patients (Hospital San Cecilio and Hospital Virgen de las Nieves) in the province of Granada (Andalusia, Spain) in two time periods: from 24 April to 20 July 2020 and from 9 December 2020 to 11 May 2021 (Supplementary Figure 1).

The inclusion criteria were active HCWs older than 20 years who were caring for COVID-19 patients, including those in all professional categories. Having a COVID-19 medical history (confirmed by PCR or serology tests) prior to the beginning of the intervention, presenting symptomology compatible with COVID-19 at the start of the intervention, being diagnosed with an immunocompromising condition, or being pregnant or planning to become pregnant in the next few months were all exclusion criteria. The study was conducted according to the Declaration of Helsinki, and the protocol was approved by the Regional Ethical Committee (Granada, Spain). Informed consent was obtained from all subjects. The trial was registered with the United States Library of Medicine under the number NCT04366180.^1^

The incidence of COVID-19 in HCWs was the primary outcome used to calculate the sample size. Few data for SARS-CoV-2 infection among HCWs were available when the protocol was set up (March 2020), so a COVID-19 incidence of 10–15% was estimated for our sample. The sample size was defined for the comparison of two independent proportions using the chi-square test. For an alpha of 5% and a power of 80%, taking 12.5% as the predicted COVID-19 incidence and 10% as the minimal difference of interest to be found between the groups and considering a probable loss of 15% of the subjects, a sample of 125 participants per group (total *n* = 250) was required.

Finally, 255 participants were found to meet the inclusion criteria and were randomly allocated to one of two groups using a computer-generated randomization procedure. The placebo group received a daily capsule containing 220 mg of maltodextrin, whereas the probiotic group received a daily capsule containing 3 × 10^9^ colony forming units of the *L. coryniformis* K8 strain in a matrix of the same maltodextrin combination in a quantity to achieve the same capsule weight (220 mg). This dose has been proven to be effective and safe in previous studies performed in adult population (12–14). The probiotic and placebo were packed in similar gelatin capsules in plastic containers, with just the randomization code distinguishing them. The individuals were given treatments for 2 months.

### Study outcomes and sample collection

The primary outcome of the study was to evaluate the incidence of SARS-CoV-2 infection confirmed by PCR or antigen testing. The secondary outcomes included determining the severity and duration of SARS-CoV-2 infection. Additionally, the immune response and side effects of the COVID-19 vaccine were evaluated in a subgroup of these HCWs.

At the baseline visit, the HCWs were informed about the study details, asked to sign the informed consent form, and received a rapid serology test for COVID-19. The subjects who were negative for this test and met the rest of the inclusion/exclusion criteria were asked about their baseline data and medical history, and the corresponding treatment was dispensed for the total duration of the study (Supplementary Figure 1). Likewise, the subjects received a data collection booklet to record their symptoms and its duration in case they had a COVID-19 infection confirmed by test. For volunteers with compatible symptomatology or in the case of close contact with a COVID-19-positive patient, a PCR or antigen test to determine SARS-CoV-2 infection was done. All COVID-19 patients continued taking the study product. The follow-up visits were conducted monthly, at which data recorded in the diary were reviewed and adverse events, defined as any unfavorable or unintended effect, were collected.

The subgroup of volunteers who received the COVID-19 vaccine during the intervention were asked for a blood sample at the end of the study. Blood was collected in Vacutainer tubes (BD Biosciences) and allowed to clot. The serum was separated within an hour by centrifugation at 1.000–1.500 × *g* for 10 min, and serum aliquots were stored at −20°C. The Liaison SARS-CoV-2 S1/S2 IgG test (DiaSorin, Antony, France), a chemiluminescent microparticle immunoassay that utilizes a mix of SARS-CoV-2 recombinant S1 and S2 proteins as capture antigens, was used to obtain quantitative measures of human SARS-CoV-2 specific IgG levels. Analyses were performed according to the instructions of the manufacturer. During the follow-up visits, information about the side effects suffered after the first and the second doses of the COVID-19 vaccine were also recorded for this subgroup of volunteers.

### Statistical analysis

Normal probability plots and the Shapiro–Wilk test were used to determine the normality of the distribution for all observed variables. Data for continuous variables are reported as the mean (standard deviation, SD) and categorical variables as n (%). Continuous variables were examined with the Student’s *t* test or the non-parametric Kruskal–Wallis technique, as applicable, for comparisons between groups at the start of the trial (probiotic group vs. control group), and categorical variables were evaluated with chi-square tests. The occurrence of SARS-CoV-2 infection was described using the incidence ratio (IR) and incidence rate ratio (IRR), with the 95% CI and *p* value for the IRR calculated by a logistic regression model adjusted by the corresponding covariates.

In the subgroup of volunteers who received the COVID-19 vaccine, data from the immunogenicity analysis are presented as the mean (SE) of the log transformed data. The sample was divided into tertiles according to the days between the first dose and the antibody analysis (cutoff points were set at ≤ 56, 57–80 and ≥ 81 days). Differences between the groups were evaluated by univariate model analysis, adjusted by the corresponding covariates. Side effects are presented as counts, percentages, and odds ratios (ORs), with the 95% CIs and *p* values for the ORs calculated by a logistic regression model adjusted by the corresponding covariates.

A general alpha level of 0.05 was used as the cutoff point for statistical significance. SPSS software version 27.0 for Windows (SPSS, Chicago, IL, United States) was used for statistical analysis.

## Results

### Study data, compliance and baseline characteristics of the subjects

**FIGURE 1:**
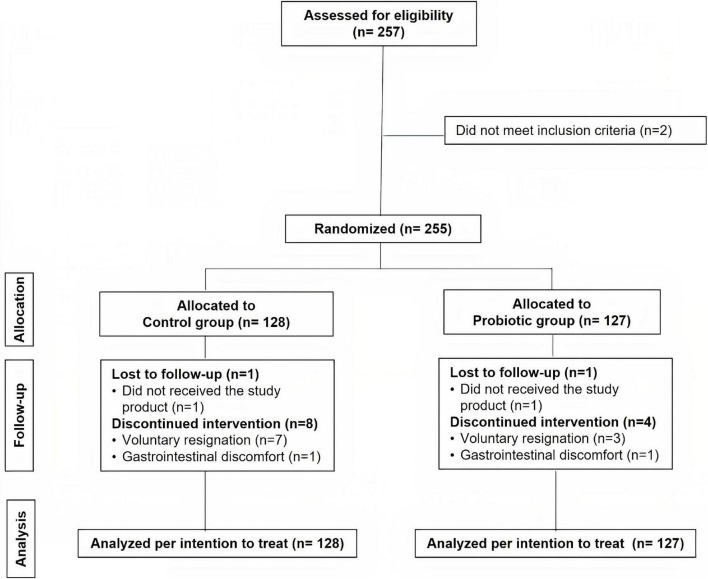
Flow chart of the study.

A total of 257 HCWs were evaluated for eligibility, of whom 2 were excluded because they did not match the inclusion criteria (Figure 1). Finally, 255 subjects were recruited and randomly assigned to two groups: the probiotic group (*n* = 127) and the control group (*n* = 128). For the reasons indicated in the study flow chart (Figure 1), nine participants in the control group and five volunteers in the probiotic group ceased the intervention and dropped out of the study before the end of the 2-month intervention period. There were no differences in the number or causes of withdrawals across the groups. The compliance percentage was corroborated to be very high (≈100%). Data were analyzed for all the subjects randomized in the study [analysis per intention to treat (ITT), *n* = 128 in the control group and *n* = 127 in the probiotic group]. No adverse events resulting from the intake of either type of treatment were reported.

Table 1 shows the baseline characteristics of the 255 HCWs included in the ITT analysis. There were no significant differences between the study groups.

**TABLE 1:** Baseline characteristics of the subjects participating in the study.

	Total (*N* = 255)	Control group (*N* = 128)	Probiotic group (*N* = 127)	*P* between groups
Age (years)	41.33 ± 11.35	41.34 ± 11.57	41.32 ± 11.18	0.993
Sex				0.325
Men	42 (16.5%)	24 (18.7%)	18 (14.2%)	
Women	213 (83.5%)	104 (81.3%)	109 (85.8%)	
BMI	24.69 ± 4.47	25.03 ± 4.99	24.35 ± 3.87	0.225
BMI classification				0.186
Normal weight	145 (56.9%)	74 (57.8%)	71 (55.9%)	
Overweight	74 (29.0%)	31 (24.2%)	43 (33.9%)	
Obese	31 (12.2%)	20 (15.6%)	11 (8.7%)	
Low weight	5 (2.0%)	3 (2.3%)	2 (1.6%)	
Smokers	61 (23.9%)	36 (28.1%)	25 (19.7%)	0.114
Dyslipidaemia	8 (3.1%)	4 (3.1%)	4 (3.1%)	0.991
Hypertension	16 (6.3%)	8 (6.3%)	8 (6.3%)	0.987
Diabetes	9 (3.5%)	3 (2.3%)	6 (4.7%)	0.303
Cardiovascular disease	5 (3.1%)	1 (0.8%)	4 (3.1%)	0.173
Chronic lung disease	5 (3.1%)	4 (3.1%)	1 (0.8%)	0.178
Past cancer disease	4 (1.6%)	2 (1.6%)	2 (1.6%)	0.994
Disease index ^1^	0.27 ± 0.61	0.29 ± 0.59	0.25 ± 0.62	0.558
Risk Factors score ^2^	0.51 ± 0.77	0.58 ± 0.80	0.45 ± 0.74	0.182

Values are mean ± SD for continuous variables and n (%) for categorical variables. *P* indicates differences between the control group and the probiotic group. ^1^Disease Index included the sum of HTA, T2DM, CVD, Lung diseases, oncology disease in the past and dyslipidemia. ^2^Risk factors score included disease index + smokers.

### COVID-19 infection incidence, severity, and duration

During the intervention, four HCWs were infected with the SARS-CoV-2 virus; two cases occurred in the control group [incidence rate (IR) (SD) = 0.016 (0.011)], and two cases occurred in the probiotic group [IR (SD) = 0.016 (0.011)]. No significant difference between the groups in the incidence of COVID-19 infection was detected [IRR (95% CI): 1.008 (0.140–7.268), *p* = 0.994]. When the model was adjusted by sex, age, hospital and the disease index, the IR (SD) in the control group was 0.0006 (1.2148) and that in the probiotic group was 0.0006 (1.1016), with no significant difference between the groups [IRR (95% CI): 0.907 (0.123–6.695), *p* = 0.923]. Of the four HCWs who were infected, one was asymptomatic, and three presented mild symptoms (cough, fever, headache, malaise, and diarrhea). The mean duration of COVID-19 symptoms was 4 ± 1 days (significant differences between the groups were not performed due to low incidence).

### Immune response to and side effects from the COVID-19 vaccine

A subgroup of 95 volunteers received the COVID-19 vaccine during the intervention (Table 2 and Supplementary Table 1). There were no significant differences in the baseline parameters between the study groups (Supplementary Table 1). Table 2 shows data related to the COVID-19 vaccine in this subgroup of subjects. All these subjects received a complete vaccination schedule of a mRNA vaccine, either the Comirnaty (BNT162b2 mRNA, BioNTech-Pfizer) or Spikevax (mRNA-1273, Moderna) vaccine, with no differences between groups (*p* = 0.127). Most of the volunteers started the intervention before receiving the first dose of the vaccine (51.6%); some started the intervention between the first and the second dose (33.7%) of the vaccine; and the least started the intervention after the second dose (12.5%) of the vaccine, with no differences between groups (*p* = 0.672). Of the 95 subjects, 85 underwent blood sample collection for specific IgG antibody analysis at the end of the study. The mean time between the first dose and the antibody analysis was 68.81 ± 23.56 days in the control group and 67.64 ± 19.78 days in the probiotic group (*p* = 0.803).

**TABLE 2:** Data related to the COVID-19 vaccine of the subgroup of subjects receiving the vaccine during intervention.

	Total vaccinated (*N* = 95)	Control group (*N* = 48)	Probiotic group (*N* = 47)	*P* between groups
Vaccine				0.127
Comirnaty	57 (60%)	33 (68.8%)	24 (51.1%)	
Spikevax	36 (37.9%)	15 (3.3%)	21 (44.7%)	
Star of the intervention				0.672
Before 1st dose	49 (51.6%)	24 (49%)	25 (51%)	
Between 1st and 2nd dose	32 (33.7%)	18 (37.5%)	14 (29.7%)	
After 2nd dose	14 (12.5%)	6 (12.5%)	8 (17%)	
Days between 1st dose and antibody analysis	68.24 ± 21.67	68.81 ± 23.56	67.64 ± 19.78	0.803

Values are mean ± SD for continuous variables and n (%) for categorical variables. *P* indicates differences between the control group and the probiotic group.

**FIGURE 2:**
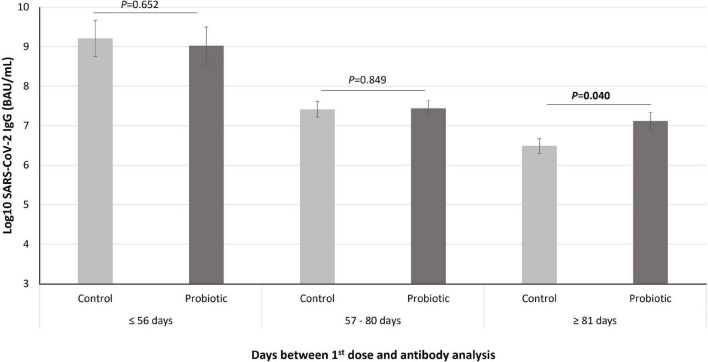
Levels of SARS-CoV-2 IgG (represented in Log10 of BAU/mL) in the subjects that received the COVID-19 vaccine during intervention (*n* = 85) divided into tertiles according to the days between the first dose and the antibody analysis. Data are represented as mean (bars) and SE (vertical lines). *P* value indicated differences between probiotic group (dark gray bars) and control (light gray bars) groups (univariate models adjusted by age, sex, age, type of vaccine, and start of the intervention).

Subjects were divided into tertiles according to the days between the first dose and the antibody analysis (cutoff points were set at ≤ 56, 57–80 and ≥ 81 days). In general, a significant difference was observed among the tertiles (*p* < 0.001), with the specific IgG levels being lower as more time passed between the first dose and the antibody analysis. However, in the subgroup of volunteers in which more than 81 days had elapsed since they received the first dose, the specific IgG levels were significantly higher in the subjects that received *L. coryniformis* K8 than in the control group (*p* = 0.040) (Figure 2).

Finally, subjects who started the consumption of the probiotic before the first dose of the vaccine reported significantly fewer side effects (any type) to the 1st dose of the vaccine (OR: 0.524, *p* = 0.043), specifically less pain at the site of inoculation (OR: 0.467, *p* = 0.017), than those who received the placebo. No significant effects were found regarding side effects to the second dose of the vaccine.

## Discussion

Health care workers are still on the front line of the battle against COVID-19 and, therefore, constituted one of the groups at the highest risk of infection during this raging pandemic (15). Vaccination has been proven to be the key for the reduction of the risk of COVID-19 infection in HCWs (16), but different studies have observed a drop in antibody levels over time (17, 18), which could have clinical consequences. Therefore, several authors have called for action to develop effective therapies and preventive measures to reduce infection and flatten the COVID-19 curve, and, in this context, probiotics could have a role (5). Our research group recently showed the usefulness of the probiotic *L. coryniformis* K8 in enhancing the immunological response of elderly people in the context of the COVID-19 vaccination (14). In the present study, we corroborated the role of *L. coryniformis* K8 as an adjuvant for boosting immunity by helping to extend the immune protection over time after COVID-19 vaccination in a group of HCWs.

Several randomized clinical trials have shown the immunomodulatory activity of the probiotic strain *L. coryniformis* K8 (12–14) and support the working hypothesis. Indeed, in one of the studies performed in the context of influenza vaccination, it was observed that the incidence of local symptoms associated with respiratory infections (cough, sore throat, and nasal congestion) was 48% lower in the group that received the probiotic strain compared to the control group in a follow-up period of 6 months (13). Interestingly, in a very recent study we performed in an elderly population in the context of the COVID-19 pandemic, we observed that the percentage of asymptomatic patients was three times higher in the group that consumed *L. coryniformis* K8 than in the control group, although we did not observe significant differences due to the low number of observed cases (IR = 0.095). However, a very low incidence of COVID-19 was detected in HCWs during the present study (IR = 0.016), so the hypothesized beneficial effect of the consumption of *L. coryniformis* K8 on the incidence and severity of COVID-19 could not be evaluated and, therefore, no conclusions can be drawn to this matter. This low COVID-19 incidence could mainly be explained by the timing of the study: the first recruitment wave (from the end of April to July 2020) was a period with a very low infection rate in Granada (Spain) (19); whereas the second recruitment wave (from December 2020 to May 2021), although coincided with the third wave of COVID-19 in Granada (Spain) (19), also concurred with the HCWs COVID-19 vaccination first campaign which was recognized and determined to be very effective (20). Therefore, further studies should be performed to reach a conclusion about the effect of *L. coryniformis* K8 on the incidence and severity of COVID-19.

We surveyed the impact of the *L. coryniformis* K8 on the immunological response elicited by the COVID-19 vaccine, observing in the vaccinated subjects a significantly lower level of specific IgG in those who had been vaccinated for the longest time. This observation is in agreement with several follow-up studies that observed a significant decline in the immune humoral response over time after a full mRNA COVID-19 vaccine schedule (18, 21–23). Interestingly, we found in the subgroup of subjects for whom more than 81 days had elapsed since they received the first dose, that the IgG-specific levels were significantly higher in the volunteers who received *L. coryniformis* K8 than in those who received the placebo. This finding is in line with the results obtained in our previous study performed with vaccinated nursing home residents. In the subset of volunteers who were diagnosed with COVID-19 during the intervention, those who took *L. coryniformis* K8 had greater IgG-specific levels than those who took the placebo (14). Although the possible mechanisms involved in the activation of IgG production must be elucidated, the obtained results may have important clinical applications, since some studies found an association between RBD-IgG levels and protection against SARS-CoV-2 in different populations (24, 25). Moreover, a very recent case–control study carried out in Sweden with more than 1.3 million people showed that vaccine efficacy waned markedly 6 months after the last dose of the COVID-19 vaccine, increasing the risk of infection, hospitalization, and severe disease (26). Additional studies with a longer follow-up time should be performed to determine the effect of *L. coryniformis* K8 on humoral immune response sustainability over time after COVID-19 vaccination.

After inoculation with any vaccine, temporary side effects caused by tissue trauma at the site of inoculation and by the activation of the immune response may appear. A study by Pormohammad et al. (27) indicated that RNA-based vaccines had a more robust immune response, exhibiting greater frequencies of reactogenicity side effects, such as site pain, redness, swelling, headache, fever, tiredness, induration, myalgia, chills, vomiting, and itching. Kadali et al. (28) also reported that after the administration of the first dose of the COVID-19 vaccine, HCWs communicated a wide range of symptoms, which, although not life-threatening, did lead to the interruption of their work activities, requiring sick leave in a percentage close to 28%. Therefore, the mitigation of the side effects after the first dose of the vaccine reported in the group that took *L. coryniformis* K8 may have repercussions both for the economy and in the quality of life of the vaccinated subjects.

Some limitations of this study must be acknowledged. First, the low COVID-19 incidence detected during the study did not allow us to fulfill the main aim of the study. Second, the sample size in the vaccinated sample subgroup was relatively small. Moreover, the study was performed in a very homogeneous population of healthy Caucasian HCWs, which limits the generalization of our results to other age groups, non-healthy population groups or ethnicities. Therefore, further studies with other population groups should be performed.

In conclusion, the administration of *L. coryniformis K8* CECT 5711 to a group of HCW helps to sustain the immune humoral response generated by the COVID-19 vaccine over time. These results support the capacity of the probiotic strain *L. coryniformis* K8 to boost the immunological response, as demonstrated in several clinical trials (12, 13), including a previous trial that was also performed in the context of the COVID-19 pandemic (14). Probiotics may be a natural and safe alternative for improving vaccination effectiveness, particularly in critical groups such as HCWs. The effect of this probiotic in the prevention and mitigation of COVID-19 should be further investigated.

## Data availability statement

The datasets presented in this article are not readily available because participants of this study did not authorize the transfer of their data to a third party or to be used for purposes other than those established in the project. Requests to access the datasets should be directed to ruth.blanco@kerry.com.

## Ethics statement

The studies involving human participants were reviewed and approved by the Regional Ethical Committee (Granada, Spain). The patients/participants provided their written informed consent to participate in this study.

## Author contributions

RR-B and MO participated in the conception of the study, designed the methodology, and contributed to the manuscript writing. JS-G participated in the design of the study and critically revised the manuscript. ÁC-V and AA recruited and followed-up the volunteers. JM-L provided study materials and performed the data curation. RB-R participated in the study design, analyzed the data, interpreted the results, and wrote the draft of the manuscript. All authors have read and approved the final manuscript.

## Footnotes

1
http://www.clinicaltrials.gov
