# Supplementary material for: Evaluation of the effect of Loigolactobacillus coryniformis K8 CECT 5711 consumption in health care workers exposed to COVID-19
Source: Front Nutr. 2022 Aug 3;9:962566. doi: 10.3389/fnut.2022.962566 (PMC9381739; doi:10.3389/fnut.2022.962566)
Supplement: Supplementary file 1 [file Data_Sheet_1.docx]

**Supplementary Material**


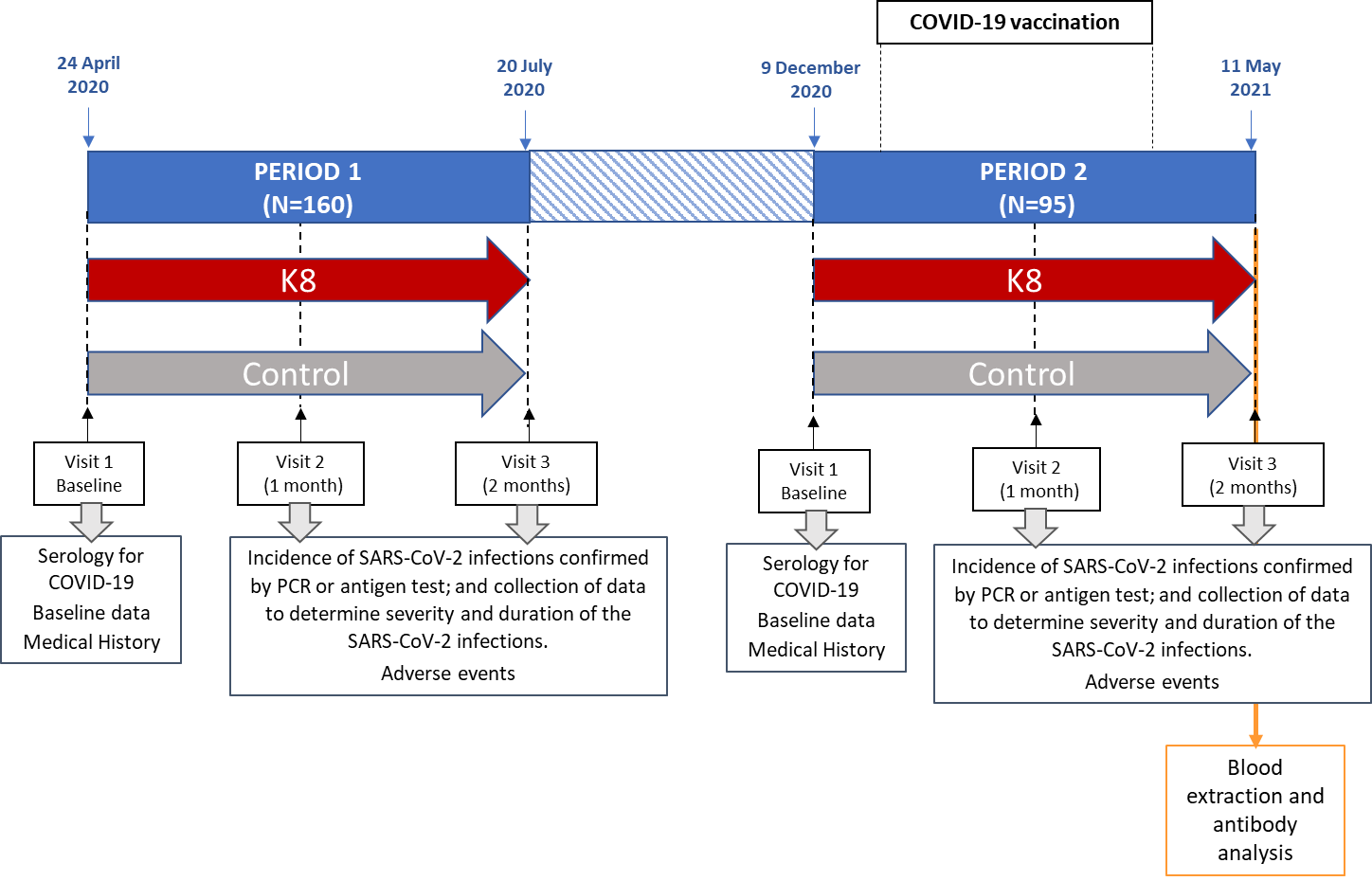


**Supplementary Figure 1**. Flow chart of study procedures

**Supplementary Table 1.** Baseline characteristics of the subgroup of subjects that received the COVID-19 vaccine during intervention

|  | Total  (N=95) | Control Group (N=48) | Probiotic Group  (N=47) | *P* between groups |
| --- | --- | --- | --- | --- |
| Age (years | 38.88±11.76 | 38.17±11.84 | 39.62±11.76 | 0.551 |
| Sex |  |  |  | 0.124 |
| Men | 13 (13.7%) | 9 (18.8%) | 4 (8.5%) |  |
| Women | 82 (86.3%) | 39 (81.3%) | 43 (91.5%) |  |
| BMI | 24.65±4.62 | 24.68±4.97 | 24.63±4.28 | 0.954 |
| BMI classification |  |  |  | 0.194 |
| Normal weight | 51(53.68%) | 27(56.25%) | 24(51.06%) |  |
| Overweight | 28(29.47%) | 10(20.83%) | 18(38.29%) |  |
| Obese | 13(13.68%) | 9(18.75%) | 4(8.51%) |  |
| Low weight | 3(3.15%) | 2(4.16%) | 1(2.12%) |  |
| Smokers | 27 (28.4%) | 14 (29.2%) | 13 (27.7%) | 0.526 |
| Dyslipidaemia | 1 (1.1%) | 1 (2.1%) | 0 (0%) | 0.505 |
| Hypertension | 7 (7.4%) | 1 (2.1%) | 6 (12.8%) | 0.052 |
| Diabetes | 2 (2.1%) | 0 (0%) | 2 (4.3%) | 0.242 |
| Cardiovascular disease | 3 (3.2%) | 0 (0%) | 3 (6.4%) | 0.117 |
| Chronic lung disease | 1 (1.1%) | 0 (0%) | 1 (2.1%) | 0.495 |
| Past cancer disease | 1 (1.1%) | 1 (2.1%) | 0 (0%) | 0.505 |
| Disease index ^1^ | 0.53±0.79 | 0.47±0.65 | 0.59±0.92 | 0.479 |
| Risk Factors score ^2^ | 0.25±0.63 | 0.18±0.44 | 0.31±0.78 | 0.315 |

Values are mean±SD for continuous variables and n (%) for categorical variables. *P* indicates differences between the control group and the probiotic group. ^1^. Disease Index included the sum of HTA, T2DM, CVD, Lung diseases, oncology disease in the past and dyslipidemia. ^2^. Risk factors score included disease index + smokers
